# Overt Versus Subclinical Hypothyroidism: An Ultrasound-Based Comparison of Thyroid Nodule Burden and Parenchymal Heterogeneity

**DOI:** 10.7759/cureus.114701

**Published:** 2026-08-17

**Authors:** Berkan Acar, Ali Muhtaroğlu, Kubilay İşsever, Elif Yilmaz, Tugrul Kesicioglu

**Affiliations:** 1 General Surgery, Giresun University, Giresun, TUR; 2 General Surgery, Özel Adatıp Hastanesi, Sakarya, TUR; 3 Internal Medicine, Giresun University, Giresun, TUR; 4 General Surgery, Özel Ordu Umut Hastanesi, Ordu, TUR

**Keywords:** hypothyroidism, overt hypothyroidism, parenchymal heterogeneity, subclinical hypothyroidism, thyroid nodule, thyroid ultrasonography

## Abstract

Background: Whether overt hypothyroidism carries a different structural thyroid profile from subclinical disease is still uncertain. We therefore compared thyroid ultrasound findings in the two groups, focusing on nodule burden and parenchymal heterogeneity.

Methods: This retrospective study included 210 adults with hypothyroidism: 53 with overt disease and 157 with subclinical disease. Demographic, laboratory, and thyroid ultrasonography data were reviewed. Ultrasound outcomes were any thyroid nodule, nodule burden, nodules ≥1 cm, cystic or solid nodules, and parenchymal echotexture. Multivariable models were adjusted for age and sex.

Results: Patients with overt hypothyroidism were older than those with subclinical hypothyroidism (median age: 52 vs. 43 years; p<0.001). Heterogeneous parenchyma was more common in patients with overt hypothyroidism than in those with subclinical hypothyroidism on unadjusted analysis (46/53 (86.8%) vs. 108/157 (68.8%); p=0.011) and remained independently associated with overt hypothyroidism after adjustment (adjusted OR: 2.94; 95% CI: 1.20-7.17; p=0.018). By contrast, the groups did not differ significantly in the prevalence of any thyroid nodule (26/53 (49.1%) vs. 71/157 (45.2%); p=0.637), nodule burden, nodules ≥1 cm, cystic nodules, or solid nodules. Age, rather than hypothyroidism category, was the main determinant of nodularity: each 10-year increase in age was associated with higher odds of any thyroid nodule (adjusted OR: 1.35; 95% CI: 1.13-1.62; p=0.001) and nodules ≥1 cm (adjusted OR: 1.59; 95% CI: 1.23-2.07; p<0.001).

Conclusion: Overt hypothyroidism was associated with more frequent parenchymal heterogeneity, but not with greater thyroid nodule burden. Thyroid nodularity and larger nodules were more closely related to age than to the hypothyroidism category.

## Introduction

The extent to which overt hypothyroidism exhibits a distinct structural thyroid phenotype compared to subclinical disease remains uncertain. This matters because hypothyroidism is common in adult practice, its burden rises with age, and it is seen more often in women [1,2]. In iodine-sufficient settings, chronic autoimmune thyroiditis underlies most cases of primary hypothyroidism, but the structural expression of thyroid failure is heterogeneous [2]. Some patients have little more than biochemical abnormality, whereas others show diffuse parenchymal change, nodularity, or both. Clarifying how these ultrasound features behave across overt and subclinical disease could help distinguish markers of disease severity from changes that accompany ageing or background thyroid pathology.

Ultrasound now occupies a central role in the structural assessment of the thyroid. It is essential for the detection of nodules and also identifies diffuse parenchymal abnormalities, which frequently indicate the underlying disease process [3]. Recent adult data suggest that such changes may appear early. Patients with subclinical-stage Hashimoto's thyroiditis have been shown to have larger thyroid glands and increased intrathyroidal vascularity even before hypothyroidism becomes manifest [4]. Structural variation has also been described within hypothyroid cohorts: in chronic thyroiditis, antibody-positive disease has been associated with larger glands and rougher internal architecture, whereas nodules and cysts were more frequent in antibody-negative hypothyroidism [5]. More severe sonographic heterogeneity and fibrosis have, in turn, been linked to greater immunological activity and higher levothyroxine requirements, and diffuse, heterogeneous parenchymal change remains one of the key ultrasound findings used to support a diagnosis of Hashimoto's thyroiditis [6,7].

Nodularity, however, may follow a somewhat different pattern. Modern imaging detects many thyroid nodules, and most are benign [3]. In a recent adult Hashimoto cohort, nodules were present in nearly one quarter of patients, with older age and larger thyroid volume independently predicting nodularity [8]. In a large contemporary health examination cohort, age and female sex again emerged as strong independent risk factors for thyroid nodules [9]. These findings suggest that nodule burden may be driven less by the hypothyroid category itself than by demographic and gland-specific factors that accumulate over time.

The remaining uncertainty pertains to how these two structural domains, diffuse parenchymal abnormality and nodularity, differ when comparing overt and subclinical hypothyroidism directly. Recent adult studies have primarily investigated autoimmune thyroiditis broadly, emphasising subclinical autoimmune stages, immunological correlates, or diagnostic accuracy. Additionally, some research has examined nodules within wider thyroid populations, but few have directly compared overt with subclinical hypothyroidism; this is inferred from the pattern of recent literature cited herein [4-9]. At the same time, the description of non-nodular thyroid parenchyma remains less standardised than the assessment of nodules [10]. Against this background, we compared thyroid ultrasound findings in adults with overt and subclinical hypothyroidism, focusing on thyroid nodule burden and parenchymal heterogeneity. We hypothesised that overt hypothyroidism would be associated with more frequent diffuse parenchymal heterogeneity, whereas nodularity would be more closely associated with age than with hypothyroidism category.

## Materials and methods

Study design and patient selection

This was a retrospective observational study conducted at Giresun University Training and Research Hospital in Giresun, Türkiye, using routinely collected clinical, biochemical, and thyroid ultrasonography data. Adult patients evaluated for hypothyroidism between October 2021 and February 2026 were screened for eligibility.

Patients were included if they were aged ≥18 years, had a documented diagnosis of either overt or subclinical hypothyroidism, had thyroid ultrasonography data adequate for structural assessment, and had sufficient laboratory data available for the evaluation of thyroid function and study variables. Patients were excluded if they were younger than 18 years, had missing or inadequate thyroid ultrasonography data, had missing laboratory data required for the study analyses, or had a history of thyroid surgery.

After application of these eligibility criteria, the final analytic cohort comprised 210 patients, including 53 with overt hypothyroidism and 157 with subclinical hypothyroidism. All primary ultrasound endpoints were complete in the analysed dataset.

Patients were analysed according to the diagnostic category assigned in the original clinical dataset. In routine endocrine practice, overt hypothyroidism is characterised by elevated serum thyroid-stimulating hormone (TSH) with reduced free thyroxine (free T4), whereas subclinical hypothyroidism is characterised by elevated TSH with free T4 within the reference range. Because this was a retrospective analysis, the original clinical classification was retained rather than reassigning cases post hoc. Serum TSH and free T4 concentrations were measured using electrochemiluminescence immunoassay-based kits on the cobas® 8000 modular analyser series, e-602 module (Roche Diagnostics, Indianapolis, Indiana, United States). The laboratory reference ranges were 0.27-4.20 mIU/L for TSH and 0.92-1.68 ng/dL for free T4. Overt hypothyroidism was characterised by an elevated TSH concentration accompanied by a free T4 concentration below the reference range, whereas subclinical hypothyroidism was characterised by an elevated TSH concentration with free T4 within the reference range.

Data collection

Data were extracted from the study dataset and reviewed for completeness and plausibility before analysis. The variables collected for the present manuscript were age, sex, levothyroxine use, TSH, free T4, anti-thyroid peroxidase (anti-TPO) antibodies, ferritin, haemoglobin, and mean corpuscular volume. A TSH/free T4 ratio was also calculated as an additional biochemical index.

The present study was designed primarily around ultrasound phenotype. The biochemical and haematological variables were therefore used to describe the cohort, to characterise the two hypothyroidism groups, and to support secondary adjusted analyses.

Thyroid ultrasonography and outcome definitions

Thyroid ultrasonography findings were obtained from the recorded imaging data. Thyroid ultrasonography examinations were performed by different operators using the same ultrasound device throughout the study period. Because of the retrospective design, the examinations were not performed or re-evaluated by a single blinded operator; therefore, inter-operator variability could not be completely excluded. The manufacturer and model of the ultrasound system were not available in the retrospective dataset.

The main structural outcomes were the presence of any thyroid nodule, overall nodule burden, the presence of at least one nodule measuring ≥1 cm, and heterogeneous thyroid parenchyma. Nodule burden was treated as an ordinal variable and classified as 0, 1, 2, or ≥3 nodules.

Additional sonographic variables included multinodular disease, defined as the presence of at least two nodules; any nodule <1 cm; and any cystic or solid nodule. Any nodule <1 cm was defined as the presence of at least one documented thyroid nodule measuring <10 mm. Nodules approximately 1-2 mm in size were generally reported in the original ultrasonography records as "millimetric nodules" without an exact numerical measurement, whereas nodules measuring ≥3 mm were recorded with their specific dimensions. Therefore, 3 mm represented the smallest explicitly quantified nodule size, although smaller millimetric nodules were also included in the <1 cm category.

Size- and composition-based variables were coded as present if a patient had at least one nodule with the relevant feature; these categories were therefore not mutually exclusive. Heterogeneous parenchyma refers to diffuse, non-nodular heterogeneity of thyroid echotexture, as described in the original ultrasound record.

Statistical analysis

The primary objective was to compare ultrasound findings between overt and subclinical hypothyroidism. Continuous variables were summarised as medians with interquartile ranges and compared using the Mann-Whitney U test. Categorical variables were summarised as counts and percentages. Binary categorical variables were compared using Fisher's exact test, whereas the four-category distribution of nodule number was compared using Pearson's chi-squared test. For the chi-squared analysis, the test statistic, degrees of freedom, and Cramér's V as the effect-size measure were reported.

To determine whether the hypothyroidism category was independently associated with structural ultrasound findings, age- and sex-adjusted regression models were fitted. Binary ultrasound outcomes, including any thyroid nodule, any nodule ≥1 cm, heterogeneous parenchyma, cystic nodule, and solid nodule, were analysed using binary logistic regression. Overall nodule burden (0, 1, 2, or ≥3 nodules) was analysed using ordinal logistic regression. Age was entered as a continuous covariate per 10-year increase, and sex was included as a covariate in all adjusted models. Effect estimates are presented as odds ratios (ORs) with 95% confidence intervals (CIs).

Secondary analyses examined selected laboratory phenotypes after adjustment for age and sex. Biomarkers with skewed distributions were analysed on a logarithmic scale and reported as adjusted geometric mean ratios, whereas haemoglobin and mean corpuscular volume were analysed on the original scale and reported as adjusted mean differences. Because parenchymal heterogeneity may be influenced by thyroid autoimmunity, an additional sensitivity model for this outcome was fitted with further adjustment for log-transformed anti-TPO. All tests were two-sided, and p-values of <0.05 were considered statistically significant. As no primary ultrasound endpoint was missing, no imputation was required for the main analyses. Statistical analyses were performed in Python (version 3.13.5, Python Software Foundation, Fredericksburg, Virginia, United States) using pandas (version 2.2.3), SciPy (version 1.17.0), and statsmodels (version 0.14.6).

Ethical considerations

The study protocol was approved by the Medical Research Ethics Committee of Giresun University (approval number: E-50877869-000-197352). Given the retrospective design and the use of existing clinical records, the requirement for informed consent was waived.

## Results

A total of 210 adults with complete ultrasound outcome data were included in the analysis, of whom 53 (25.2%) had overt hypothyroidism and 157 (74.8%) had subclinical hypothyroidism. Patients with overt hypothyroidism were older than those with subclinical hypothyroidism (median age: 52.0 (IQR: 44.0-65.0) vs. 43.0 (IQR: 27.0-55.0) years; p<0.001), whereas the proportion of women was similar between the two groups (39/53 (73.6%) vs. 110/157 (70.1%); p=0.727). As expected, the overt hypothyroidism group had higher TSH concentrations, lower free T4 concentrations, and a higher TSH/free T4 ratio. Ferritin and haemoglobin levels were also lower in patients with overt hypothyroidism, whereas anti-TPO levels, mean corpuscular volume, and levothyroxine use did not differ significantly between the groups (Table 1).

**Table 1:** Clinical and laboratory characteristics by hypothyroidism category Values are presented as median (interquartile range) or n (%). P-values were derived using the Mann-Whitney U test for continuous variables and Fisher's exact test for categorical variables. TSH: thyroid-stimulating hormone; free T4: free thyroxine; anti-TPO: anti-thyroid peroxidase; MCV: mean corpuscular volume; IQR: interquartile range

Characteristic	Overall (n=210)	Overt (n=53)	Subclinical (n=157)	P-value
Age, years	46.5 (30.0-58.0)	52.0 (44.0-65.0)	43.0 (27.0-55.0)	<0.001
Female sex	149 (71%)	39 (73.6%)	110 (70.1%)	0.727
Any levothyroxine use	53 (25.2%)	11 (20.8%)	42 (26.8%)	0.466
TSH, mU/L	6.3 (5.2-8.9)	8.6 (6.1-11.5)	5.9 (5.1-8.0)	<0.001
Free T4, ng/dL	1.0 (0.9-1.1)	0.8 (0.7-0.9)	1.1 (1.0-1.2)	<0.001
TSH/free T4 ratio	6.3 (4.8-9.5)	10.7 (7.3-15.0)	5.6 (4.7-7.6)	<0.001
Anti-TPO, IU/mL	26.5 (11.0-181.4)	43.0 (12.0-206.0)	23.0 (11.0-153.2)	0.323
Ferritin, µg/L	45.5 (23.0-95.8)	33.0 (18.0-59.0)	57.0 (26.0-102.0)	0.012
Haemoglobin, g/dL	13.4 (12.7-14.3)	13.3 (12.3-13.8)	13.6 (12.7-14.5)	0.016
MCV, fL	87.0 (83.2-90.0)	87.0 (83.0-90.0)	87.0 (84.0-89.0)	0.617

In the unadjusted analysis of thyroid ultrasonography findings, heterogeneous parenchyma was more common in patients with overt hypothyroidism than in those with subclinical hypothyroidism (46/53 (86.8%) vs. 108/157 (68.8%); p=0.011). By contrast, the prevalence of any thyroid nodule did not differ significantly between the groups (26/53 (49.1%) vs. 71/157 (45.2%); p=0.637). No significant between-group differences were observed in multinodular disease, nodules <1 cm, nodules ≥1 cm, cystic nodules, or solid nodules. The distribution of total nodule number was also similar between the groups (Pearson's χ²(3)=0.507; p=0.917; Cramér's V=0.049) (Table 2).

**Table 2:** Thyroid ultrasound findings by hypothyroidism category Values are presented as n (%). Binary categorical variables were compared using Fisher's exact test. The p-value for the distribution of nodule number refers to the overall four-category comparison using Pearson's chi-squared test (χ²(3)=0.507; p=0.917; Cramér's V=0.049).

Ultrasound finding	Overall (n=210)	Overt (n=53)	Subclinical (n=157)	P-value
Any thyroid nodule	97 (46.2%)	26 (49.1%)	71 (45.2%)	0.637
Multinodular disease (≥2 nodules)	52 (24.8%)	15 (28.3%)	37 (23.6%)	0.581
Any nodule <1 cm	92 (43.8%)	24 (45.3%)	68 (43.3%)	0.873
Any nodule ≥1 cm	32 (15.2%)	11 (20.8%)	21 (13.4%)	0.268
Solid nodule	13 (6.2%)	1 (1.9%)	12 (7.6%)	0.192
Cystic nodule	60 (28.6%)	18 (34%)	42 (26.8%)	0.379
Heterogeneous parenchyma	154 (73.3%)	46 (86.8%)	108 (68.8%)	0.011
Number of nodules: 0	114 (54.3%)	27 (50.9%)	87 (55.4%)	0.917
Number of nodules: 1	44 (21%)	11 (20.8%)	33 (21%)	-
Number of nodules: 2	14 (6.7%)	4 (7.5%)	10 (6.4%)	-
Number of nodules: ≥3	38 (18.1%)	11 (20.8%)	27 (17.2%)	-

**Figure 1:**
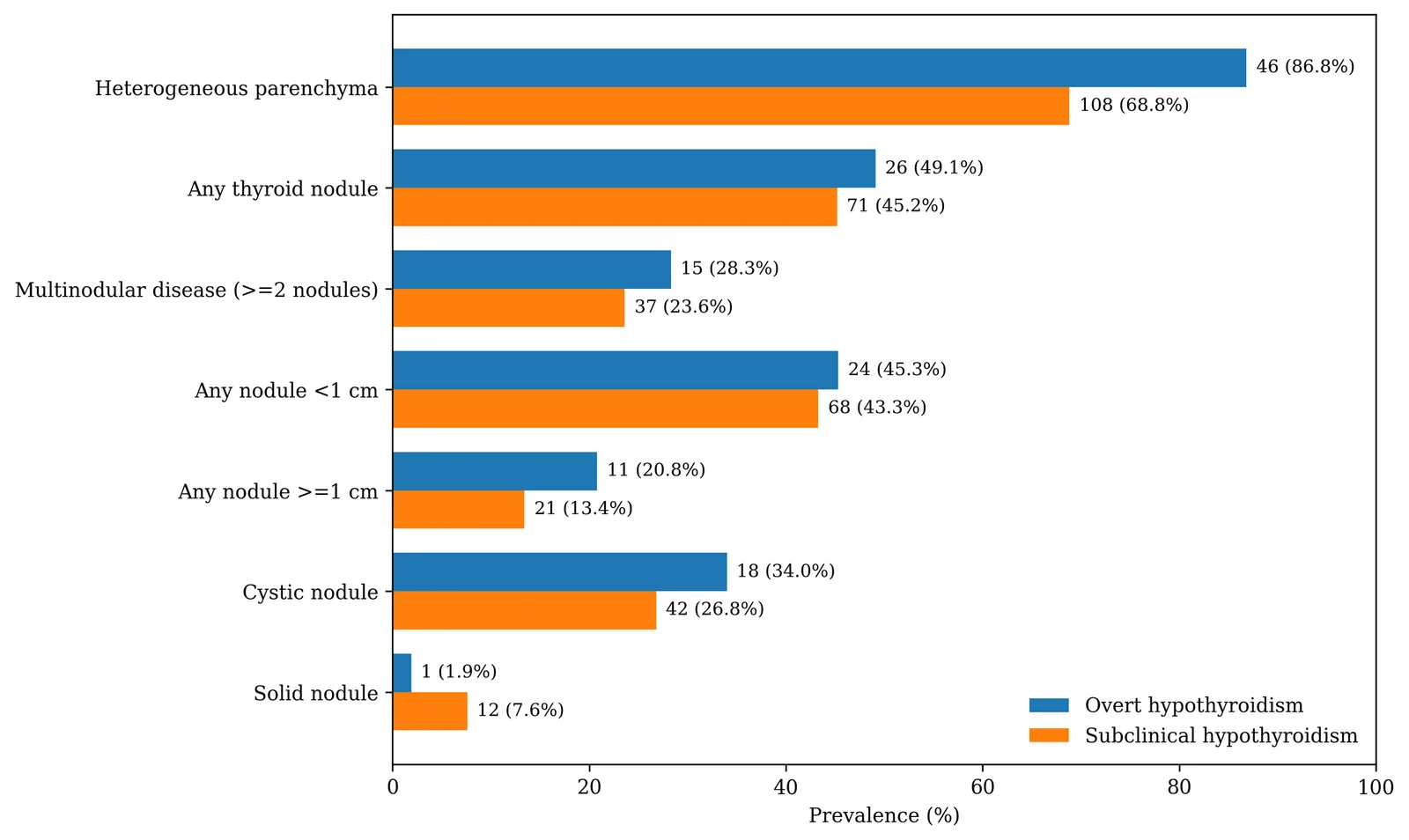
Age- and sex-adjusted odds ratios for overt versus subclinical hypothyroidism across the main structural ultrasound outcomes Squares indicate adjusted OR, and horizontal lines indicate 95% CI. The dashed vertical line marks an odds ratio of 1.0.

In age- and sex-adjusted analyses, overt hypothyroidism remained independently associated with heterogeneous parenchyma (adjusted OR: 2.94; 95% CI: 1.20-7.17; p=0.018). No independent association was seen between hypothyroidism category and any thyroid nodule, greater nodule burden, nodules ≥1 cm, cystic nodules, or solid nodules (Table 3, Figure 1). Age, rather than hypothyroidism category, emerged as the clearest determinant of nodularity: each 10-year increase in age was associated with higher odds of any thyroid nodule (adjusted OR: 1.35; 95% CI: 1.13-1.62; p=0.001), a greater overall nodule burden (adjusted OR: 1.35; 95% CI: 1.14-1.60; p<0.001), and nodules ≥1 cm (adjusted OR: 1.59; 95% CI: 1.23-2.07; p<0.001). Female sex was not independently associated with the main structural ultrasound outcomes.

**Table 3:** Age- and sex-adjusted models for structural ultrasound outcomes Binary ultrasound endpoints were analysed with logistic regression. Nodule burden was analysed with ordinal logistic regression. Age was modelled per 10-year increase.

Outcome	Predictor	Adjusted OR	95% CI	P-value
Any thyroid nodule	Overt vs. subclinical hypothyroidism	0.83	0.42-1.62	0.582
	Age (per 10 years)	1.35	1.13-1.62	0.001
	Female sex	1.18	0.63-2.20	0.608
Nodule burden (0/1/2/≥3 nodules)	Overt vs. subclinical hypothyroidism	0.86	0.46-1.61	0.641
	Age (per 10 years)	1.35	1.14-1.60	<0.001
	Female sex	1.03	0.58-1.84	0.907
Any nodule ≥1 cm	Overt vs. subclinical hypothyroidism	1.09	0.46-2.57	0.847
	Age (per 10 years)	1.59	1.23-2.07	<0.001
	Female sex	1.67	0.67-4.17	0.269
Heterogeneous parenchyma	Overt vs. subclinical hypothyroidism	2.94	1.20-7.17	0.018
	Age (per 10 years)	1.03	0.85-1.25	0.794
	Female sex	0.74	0.36-1.50	0.399
Cystic nodule	Overt vs. subclinical hypothyroidism	1.15	0.57-2.32	0.701
	Age (per 10 years)	1.20	1.00-1.46	0.056
	Female sex	1.02	0.52-2.00	0.953
Solid nodule	Overt vs. subclinical hypothyroidism	0.17	0.02-1.37	0.095
	Age (per 10 years)	1.35	0.95-1.91	0.093
	Female sex	1.05	0.30-3.64	0.938

In age- and sex-adjusted secondary analyses, overt hypothyroidism remained associated with a more pronounced biochemical hypothyroid profile, including higher TSH (geometric mean ratio: 1.36; 95% CI: 1.19-1.56; p<0.001), lower free T4 (geometric mean ratio: 0.75; 95% CI: 0.70-0.80; p<0.001), and a higher TSH/free T4 ratio (geometric mean ratio: 1.83; 95% CI: 1.55-2.14; p<0.001). Ferritin was 40% lower in overt hypothyroidism after adjustment (geometric mean ratio: 0.60; 95% CI: 0.45-0.80; p<0.001), and haemoglobin remained lower by an adjusted mean difference of -0.51 g/dL (95% CI: -0.92 to -0.09; p=0.017). There were no adjusted between-group differences in anti-TPO or mean corpuscular volume (Table 4).

**Table 4:** Age- and sex-adjusted differences in laboratory phenotypes Skewed biomarkers were analysed on the logarithmic scale and are reported as adjusted geometric mean ratios. Haemoglobin and MCV are reported as adjusted mean differences. TSH: thyroid-stimulating hormone; free T4: free thyroxine; anti-TPO: anti-thyroid peroxidase; MCV: mean corpuscular volume

Laboratory phenotype	Effect measure	Estimate	95% CI	P-value
TSH, mU/L	Adjusted geometric mean ratio	1.36	1.19-1.56	<0.001
Free T4, ng/dL	Adjusted geometric mean ratio	0.75	0.70-0.80	<0.001
Anti-TPO, IU/mL	Adjusted geometric mean ratio	1.26	0.75-2.10	0.381
TSH/free T4 ratio	Adjusted geometric mean ratio	1.83	1.55-2.14	<0.001
Ferritin, µg/L	Adjusted geometric mean ratio	0.60	0.45-0.80	<0.001
Haemoglobin, g/dL	Adjusted mean difference	-0.51	-0.92 to -0.09	0.017
MCV, fL	Adjusted mean difference	-0.42	-2.25 to 1.41	0.650

Given that parenchymal heterogeneity may partly indicate thyroid autoimmunity, a sensitivity analysis was conducted with additional adjustment for log-transformed anti-TPO levels. The relationship between overt hypothyroidism and heterogeneous parenchyma remained statistically significant (adjusted OR: 2.85; 95% CI: 1.14-7.12; p=0.024).

## Discussion

This study set out to answer a practical clinical question: when hypothyroidism is overt rather than subclinical, does the thyroid look more nodular on ultrasound or simply more diffusely abnormal? Our findings support the latter interpretation. Heterogeneous parenchyma was observed in 46 of 53 patients (86.8%) with overt hypothyroidism and in 108 of 157 patients (68.8%) with subclinical hypothyroidism. This association persisted after adjustment for age and sex and remained significant after additional adjustment for anti-TPO levels in the sensitivity analysis. By contrast, the prevalence of any thyroid nodule was similar between patients with overt and subclinical hypothyroidism (26/53 (49.1%) vs. 71/157 (45.2%)). Age, rather than hypothyroidism category, was the factor most consistently linked to nodularity. In other words, the structural distinction between overt and subclinical hypothyroidism in this cohort lay mainly in the background gland rather than in focal nodular disease.

Most recent studies have examined autoimmune thyroiditis across its spectrum or focused on early autoimmune stages, rather than directly comparing overt and subclinical hypothyroidism head-to-head [6,11,12]. Within that literature, the excess of parenchymal heterogeneity in overt disease is biologically plausible. In a 2022 adult case-control study, Ünal et al. showed that heterogeneity, hypoechogenicity, and pseudonodular change were closely related to inflammatory activity and that hypothyroid patients had more heterogeneous and hypoechoic parenchyma than euthyroid patients [11]. Kenarlı et al. later reported that moderate-to-high heterogeneity and fibrosis were associated with higher anti-TPO levels and greater levothyroxine requirements [6]. Read alongside our findings, these data suggest that sonographic heterogeneity likely reflects the cumulative structural consequences of autoimmune injury and tissue remodelling, which become more apparent as thyroid failure progresses. That said, our data do not allow a direct histopathological explanation, so this interpretation should remain measured rather than definitive.

The subclinical group, however, was far from structurally normal. Heterogeneous parenchyma was already common in subclinical hypothyroidism, which fits with the view that ultrasound abnormalities can emerge before full biochemical decompensation. Angelopoulos et al., studying patients in the subclinical stages of Hashimoto's thyroiditis, found that larger thyroid size and increased intrathyroidal vascularity may be present even before overt hypothyroidism develops [13]. Additional evidence supports the complementary role of thyroid ultrasonography in evaluating diffuse and focal structural abnormalities in hypothyroidism. Wakita et al. demonstrated that quantitatively assessed thyroid parenchymal heterogeneity was positively correlated with anti-TPO levels in patients with Hashimoto's thyroiditis, suggesting that sonographic heterogeneity may reflect underlying autoimmune activity and thyroid tissue injury [14]. In a prospective study of patients with primary hypothyroidism without clinically suspected nodular goitre, García González and García Pascual reported that ultrasonography identified chronic thyroiditis in some patients with negative thyroid autoantibodies and also detected clinically unsuspected thyroid nodules [15]. These observations reinforce the value of ultrasonography as a complementary structural assessment tool; however, our findings indicate that overt hypothyroidism itself should not be interpreted as evidence of a greater focal nodule burden. The most sensible way to interpret the present data is therefore as a continuum rather than a hard split: diffuse parenchymal change may begin early, but it becomes more common once hypothyroidism is overt.

The nodule findings are just as instructive. The absence of a meaningful difference in nodule burden between overt and subclinical hypothyroidism argues against the assumption that more severe biochemical hypothyroidism necessarily translates into a more nodular gland. Recent adult cohorts point in the same direction. In the Egyptian Hashimoto cohort reported by El Aghoury et al., older age and larger thyroid volume were major predictors of nodularity, while nodular presentation was shaped more by patient and gland characteristics than by a simple distinction between milder and more advanced hypothyroid states [8]. In a much larger health examination study of more than 23,000 adults, Yu et al. likewise found that thyroid nodule prevalence rose steadily with age and that older age and female sex were independent predictors [9]. Our adjusted models sharpen that message for the present clinical setting: once age and sex were taken into account, the hypothyroidism category was not associated with any nodule, greater nodule burden, or nodules ≥1 cm, whereas age remained consistently associated with all three outcomes.

The lack of an independent sex effect in our models probably reflects the composition of this cohort more than a genuine absence of sex-related risk. Women predominated in both groups, and the comparison was disease-specific rather than population-based. In that setting, the age signal may simply be easier to detect than the sex signal. Clinically, the more important point is that the biochemical category itself contributed very little to nodule prediction once age was considered.

The anti-TPO sensitivity analysis is also worth noting. Recent studies confirm that serology and sonography are related, but not interchangeable, markers of autoimmune thyroid disease. Iwamoto et al. showed that antibody-positive hypothyroid patients had significantly larger thyroid glands than antibody-negative patients [5]. Tan et al., in a large cross-sectional study, found that both ultrasound and thyroid autoantibodies have imperfect standalone diagnostic performance for Hashimoto's thyroiditis, despite good specificity [7]. Against that background, the persistence of the association between overt hypothyroidism and heterogeneous parenchyma after adjustment for anti-TPO suggests that ultrasound captures a structural dimension of disease related to, but not wholly explained by, circulating antibody levels. That is clinically useful because it means the sonographic background gland may carry information beyond serology alone.

There is a straightforward practical implication. Nodules should be assessed based on their own ultrasound characteristics and size criteria, not on whether hypothyroidism is overt or subclinical. Current European guidance on thyroid nodules is explicitly ultrasound-based in its approach to risk stratification and management [4]. Our findings sit comfortably with that framework: overt hypothyroidism may increase the likelihood of diffuse parenchymal abnormality, but it does not appear to justify a lower threshold for nodule-driven investigation. At the same time, recent work by Trimboli and Bojunga has highlighted how poorly standardised the description of non-nodular thyroid parenchyma still is compared with nodule assessment [10]. Our study adds to the case for paying closer attention to the background gland, because parenchymal heterogeneity seems to distinguish overt from subclinical hypothyroidism more clearly than nodule metrics do.

The secondary biochemical differences mainly serve as an internal check that the clinical categories were behaving as expected. After adjustment, the overt group retained the more pronounced biochemical hypothyroid profile and had lower ferritin and haemoglobin levels. We would be cautious about overemphasising these secondary differences in an ultrasound-focused paper, but they do reinforce that the two groups were biologically distinct, even though the main structural divergence remained confined to diffuse parenchymal change rather than nodularity.

Study limitations

Several limitations deserve emphasis. The study was retrospective and cross-sectional, so it cannot establish whether the ultrasound pattern seen in subclinical disease evolves longitudinally into the phenotype seen in overt hypothyroidism. The overt subgroup was modest in size, which inevitably widens confidence intervals for less common outcomes such as solid nodules. Ultrasound variables were based on recorded clinical assessments rather than central blinded re-reading. Although the same ultrasound device was used throughout the study period, examinations were performed by different operators; therefore, inter-operator variability and associated measurement bias cannot be completely excluded. In addition, parenchymal heterogeneity was captured as a pragmatic binary variable rather than on a graded scale. Histopathological confirmation was not available, and anti-TPO cannot fully summarise autoimmune activity. More broadly, the field is only now moving towards a more standardised language for non-nodular thyroid ultrasound patterns, which remains an important methodological challenge. Even so, the study also has clear strengths: complete primary ultrasound endpoints, a direct comparison between overt and subclinical hypothyroidism within the same cohort, multivariable adjustment for the main demographic confounders, and a sensitivity analysis showing that the parenchymal signal persisted after accounting for anti-TPO.

## Conclusions

Overall, these findings suggest that overt hypothyroidism is distinguished from subclinical disease chiefly by a more heterogeneous thyroid background, not by a heavier thyroid nodule burden. That is a modest conclusion, but a useful one. It separates diffuse parenchymal change from nodularity, suggests that age is a stronger determinant of nodules than hypothyroidism category, and supports a more nuanced reading of thyroid ultrasound in patients with hypothyroidism. Prospective studies with longitudinal follow-up and standardised parenchymal scoring would now be valuable in determining whether worsening heterogeneity tracks progression from subclinical to overt disease more reliably than nodule-based measures do.
